# Cognitive reflection, 2D:4D and social value orientation

**DOI:** 10.1371/journal.pone.0212767

**Published:** 2019-02-22

**Authors:** Kobe Millet, Aylin Aydinli

**Affiliations:** Department of Marketing, Vrije Universiteit Amsterdam, Amsterdam, Netherlands; Middlesex University, UNITED KINGDOM

## Abstract

The current study seeks confirmation for the hypothesis that 2D:4D (positively) predicts prosociality when people are more likely to rely on intuition than deliberation. We assess intuition and deliberation using the Cognitive Reflection Test (CRT) and measure prosociality via the validated Social Value Orientation (SVO) slider measure. Although our results do not provide collective evidence for our main proposition, we observe in the data that for low (right) 2D:4D men, the more intuitive they are, the less prosocial they become, whereas for high (right) 2D:4D men the thinking style does not affect their prosociality. Importantly, we find that two alternative measures of cognitive reflection, CRT and CRT-2, differently relate to prosocial decision making such that only CRT-2 (but not the classic CRT) positively predicts prosociality. Given that previous research on the role of cognitive reflection and 2D:4D in prosocial decision making provided inconsistent results, the present study findings are highly valuable to get a better understanding in this domain of study. Furthermore, some of our findings invite further confirmatory tests, thereby opening up multiple avenues for further research.

## Introduction

Second to fourth digit ratio is the ratio of the index (2D) to ring (4D) finger, shortly referred to as 2D:4D. It is a putative marker of prenatal exposure to testosterone [1, 2] with a lower ratio pointing towards exposure of higher testosterone levels during pregnancy. Direct evidence for 2D:4D as a biomarker for organizational effects of prenatal testosterone has been provided in non-human mammals like rats [3] and mice [4, 5] and much more (indirect) evidence in humans has been provided as well. One of the most robust effects is the sexual dimorphism in 2D:4D with men having lower digit ratios than women [6], in line with the observation that testosterone levels in amniotic fluid are higher for male than female fetuses [7, 8]. At least, as illustrated by the increasing number of publications in the last decades 2D:4D is commonly accepted as an indirect biomarker for fetal testosterone exposure (despite a need for further validation [9]).

Over the years, evidence has been provided for the relation between 2D:4D and a multitude of personality traits, decisions and behavior, both in the lab and the field. For instance, 2D:4D has been associated with sex role identity [10], depression [11], aggression [12], dominance [13, 14], risk taking [15], social preferences [16, 17], achievement in sports [18, 19] and cognitive tests [20], product preferences [21], gift giving [22], etc. However, there is uncertainty about which relationships can be consistently replicated [23, 24]. To our interest, the relation between 2D:4D and social preferences has been doubted [25]. Given the paucity of clear evidence in the field, it is important to continue the investigation of the association between 2D:4D and specific personality traits, decisions and behavior to get more insight into reliable relationships.

One perspective that aims to clarify seemingly inconsistent results in 2D:4D research highlights the importance of the specific study context [23]. For instance, one interpretation of the inconsistencies in research findings on the association between 2D:4D and risk taking, aggression and dominance focuses on the potential role of the status relevance of the behavior at hand [26]. It has been suggested that some contexts (e.g. sports competition) entail status enhancing behaviors which influence the relationship between 2D:4D and specific aggressive, risky or dominant decisions [26]. At least, whereas previous research has typically focused on direct relationships, a contextual perspective suggests that it may be fruitful to give more attention to potential interaction effects focusing on specific contextual characteristics that may influence the relationship between 2D:4D and other variables. Therefore, the present research focuses on prosocial decision making and investigates a potential moderator that is theoretically meaningful in studying the relationship between 2D:4D and prosociality.

Previous research on the effect of 2D:4D on prosociality yields mixed results. For instance, low 2D:4D has been related to increased cooperation levels in public good [17] and dictator [27] type of games. On the other hand, there is also some evidence for low 2D:4D and decreased prosociality [28–30], albeit this may depend on the particular context of the decision [23, 28] or the incentivization of decisions [26]. Finally, in other type of games and contexts, some null results and curvilinear relationships have been reported as well [16, 31, 32]. Given this multitude of studies with different paradigms, it is still unclear how 2D:4D is actually related to prosociality, suggesting the importance of studying potential moderators. As such, we focus on a variable that may be of importance to understand the relation between 2D:4D and prosociality: cognitive reflection (i.e. intuitive vs deliberate processing).

### Cognitive reflection as a potential moderator between 2D:4D and prosociality

Alonso and colleagues [33] observe that low 2D:4D is related to less generous and more selfish behavior in a multiple-rounds dictator-type of economic game when people are already in a disadvantaged position (i.e. worst paid agent) and when they score low on the cognitive reflection test (hereafter CRT). CRT, introduced by Frederick [34], consists of a set of numerical problems that all have an intuitive, yet incorrect, answer that may be selected by those who do not reflect carefully enough. Therefore, CRT scores reflect people’s ability to resist reliance on intuition in favour of reliance on deliberation. The question arises whether low 2D:4D people have a tendency to be less prosocial when they rely on intuitive (vs. deliberative) decision making. At least, some findings in the literature appear to be in line with this proposition. Some evidence shows that low male 2D:4D is only related to lower prosociality and increased selfishness in the dictator position when aggression has been primed [27]. Interestingly, the mere interaction of men with a gun [35] as well as exposure to an aggressive video [36] leads to an increase of circulating testosterone levels and it has been shown that higher testosterone levels decrease men’s performance on a cognitive reflection test [37]. Thus, the moderating effect of the aggression prime may not necessarily be driven by the aggressive content of the prime, but possibly by its effect on cognitive reflection. Further, a positive relationship has been shown between young (resp. 11- and 6-to-9—year old) children’s 2D:4D and prosociality in different resource allocation paradigms [28, 29]. As it has been shown that young children have lower impulse control than adults [38], this is again consistent with the idea that reliance on intuition (vs. deliberation) may drive the relationship between low 2D:4D and selfish preferences. Overall these findings suggest that part of the inconsistencies in literature could be explained by the differences in the extent to which people’s decisions are based on intuition vs. deliberation.

More specifically, we will test the hypothesis that 2D:4D positively predicts prosociality when people rely on intuition, but not when they think more deliberately. That is, we predict that the lower 2D:4D the less prosocial people become when they score low on cognitive reflection. In contrast, for people scoring high on cognitive reflection, we do not expect that 2D:4D has any effect on prosociality. To do so, we will explore the interaction between 2D:4D and cognitive reflection on prosociality, as measured by the social value orientation (SVO) slider measure (see Method section below for a clarification of this specific measurement). Social value orientation (SVO) is the prevalent conceptualization of social preferences in psychology [39] and has originally been defined as a personal characteristic of how people interact in social dilemmas [40]. With the original SVO measure, people are typically categorized as either prosocial or proself. The slider measure we make use of is a relatively recently developed, more finegrained, continuous measure of prosociality and opens opportunities to use as a dependent variable [41].

### Cognitive reflection and prosociality

While the main focus of our study is on the potential moderating role of cognitive reflection on the relation between 2D:4D and prosociality, our findings may also provide insight into the direct relationship between cognitive reflection and prosociality. On the one hand, previous research suggests that prosociality is an intuitive response, whereas deliberation may lead to more selfish decisions [42–45]. Some studies identified moderators for this effect showing prosociality is an intuitive response in women (as opposed to men) [46] and for people with (as opposed to without) experience in economic games [47]. Others again provide evidence that deliberation leads to more selfish decisions in men (but not women) [48]. Still, it is unclear if people really act in accordance with this so-called ‘social heuristic hypothesis’ [49]. On the other hand, a reflective model of prosociality claims that we need to overcome our prepotent, selfish impulses [45, 50]. And again, there is consistent evidence with this perspective showing, for example, that automatic, spontaneous reactions in one-shot anonymous prisoner’s dilemma games tend to be egostic [51]. Overall, the discussion is not finished yet and therefore it is interesting by itself to explore if there is a direct relationship (and if so, how this relationship looks like) between cognitive reflection and our SVO measure.

In our empirical study, we adopt two measures of cognitive reflection, the 3-item classic cognitive reflection test [34] as well as an alternate, recently developed, 4-item cognitive reflection test [52]. Whereas the original CRT has been widely adopted as a measurement of cognitive reflection, it has recently been criticized as relying too much on numeracy skills [53] and behaving differently than the CRT-2 [54]. By adopting two CRT measures, not only we are able to test our main interaction hypothesis and the direct relationship between cognitive reflection and prosociality separately for each of the measures, but we also gain more insight into potential differences between CRT and CRT-2.

Finally, we adopted the extended version of the (non-incentivized) SVO slider measure [42] as it is not only a validated measurement of prosociality, but also because the additional items in the extended version are specifically set up to identify to what extent the aim to maximize collective outcomes (‘joint maximization’) vs. the aim to minimize differences between oneself and the other (‘inequality aversion’) drive prosociality. By using this extended version, the current study may provide the opportunity to explore potential relationships between inequality aversion vs. joint gain maximization, 2D:4D and cognitive reflection. A recent study [55] provides some evidence in a group context that low CRT scores are associated with individuals’ concerns for their relative shares (i.e. distribution of shares among group members) and high CRT scores are associated with individuals’ concerns for social efficiency (i.e. total level of group resources). Following these results we would expect that increased cognitive reflection is positively related to the maximization of joint gains and negatively related to inequality aversion.

## Method

Two hundred eighteen participants between 18 and 31 years of age took part in the study (127 men, 91 women) of which 167 in return for course credit and 51 in return for a hedonic food reward (chocolate or pringles). All subjects gave informed consent in accordance with the Declaration of Helsinki and the study was approved by the School of Business and Economics Research Ethics Review Board (Vrije Universiteit Amsterdam). The study took place in the experimental lab of the School of Business and Economics where every participant was assigned to a computer in a partially enclosed carrel in which they could not see one another and could not talk. A maximum of 14 students participated at the same time.

First, participants received 11 questions to answer, seven of which were the test items of two cognitive reflection tests: the 3-item CRT [34] and 4-item CRT-2 [52]. The CRT items were intermixed in random order and the remaining four decoy questions [52] were presented at fixed positions inbetween (2^nd^; 4^th^; 7^th^; 9^th^). For each subject, two separate total general cognitive reflection scores were computed by summing up the relevant items of the CRT and CRT-2. A total general cognitive reflection score ranges from 0 (intuitive decision making) to 3 (deliberate decision making) for the 3-item CRT and from 0 (intuitive decision making) to 4 (deliberate decision making) for the 4-item CRT-2. We measured the CRT items first as it has been showed that students perform better in the beginning than at the end [56]. Thereafter, participants completed the extended (15-item) Social Value Orientation Slider Measure [39, 41]. This measure exists of 15 different resource allocation dilemmas in which participants indicate how they would distribute monetary resources between themselves and another anonymous person. The responses to the six primary items determine a participant’s SVO angle, where a higher value indicates higher pro-sociality. The main analyses will adopt the continuous SVO angle as the dependent measure. We discuss the remaining secondary items of the SVO slider measure in the supplementary materials.

Finally, both left and right hands were scanned to measure finger lengths. Participants placed their hand palms on the glass plate of a scanner and finger lengths were measured by two independent raters (by means of Adobe Photoshop) from the ventral proximal crease to the tip of the finger. When there was a band of creases at the base of the digit finger lengths were measured from the bottom crease. Both raters were asked to indicate any unclarity for each specific finger measurement (i.e. resp. left and right index and ring fingers). After they measured all fingers, both raters were requested to measure the right or left index and ring fingers again (a) when one of the raters gave a comment on a specific measurement and/or (b) the difference in 2D:4D between both raters was larger than.04. Moreover, raters were asked to provide comments on each of the measurements they were asked to measure again. If deemed necessary, a rater could advise not to include a specific 2D:4D measurement (because the creases were not clear, etc.). If one of the raters recommended not to include a specific data point, the participant was (only) excluded from the specific analysis in which the ratio was included. Further, data points were also excluded of those ratios in which one of the raters indicated it was not possible to clearly measure one of both finger lengths. Consequently, 8 data points were excluded from the data analyses that included left 2D:4D and 12 data points were excluded from the analyses that included right 2D:4D. Assessments of both raters were highly correlated (resp. *r*_*left 2D*:*4D*_ = .93 and *r*_*right 2D*:*4D*_ = .87), speaking towards the accuracy of the measurements. Therefore, we averaged both assessments for left 2D:4D as well as for right 2D:4D to attain single left and right 2D:4D measures. Only these averaged 2D:4Ds are used in the statistical analyses. Consistent with the prior literature, we treat left and right 2D:4D measures separately in our subsequent statistical analyses and focus on right 2D:4D in our main text as this has been considered a better indicator of prenatal androgenisation than left-hand 2D:4D [6]. For the sake of completeness, we include results pertaining to left 2D:4D in the supplementary materials (see S1 Table).

## Results

We first explored sex differences (see Table 1) in (a) left and right 2D:4D (b) both CRT and CRT-2 and (c) SVO angle. In line with previous literature, men have a lower 2D:4D [6], score higher on the CRT [34, 52] and turn out to be less pro-social than women [57]. We further replicate the findings indicating that there is no sex difference on the CRT-2 [52] and that the right 2D:4D sex difference is larger than the left 2D:4D sex difference [6]. Next, we performed simple correlation analyses in men and women separately between left 2D:4D, right 2D:4D, CRT, CRT-2, and SVO angle (see Table 2a and 2b). Interestingly, CRT and CRT-2 tend to differ in their relation with the general SVO angle measurement. A correlational analysis (irrespective of gender) indicates a positive relationship between SVO angle and the CRT-2 (*r* = .133; *p* = .050), but not the CRT (*r* = -.031, *p* = .649).

**Table 1:** Sex differences on the different measurements.

	Males			Females			Sex Difference	
	*N*	*M*	*SD*	*N*	*M*	*SD*	*t*	*p*
Right 2D:4D	121	.9574	.0289	85	.9720	.0329	3.35	.001
Left 2D:4D	123	.9617	.0267	87	.9698	.0283	2.09	.038
CRT	127	1.91	1.04	91	1.31	1.08	4.13	.0001
CRT-2	127	2.49	1.04	91	2.29	.99	1.45	.149
SVO angle	127	22.47	14.17	91	27.58	11.15	2.88^a^	.003

^a^as variances are not equal, equal variances are not assumed in the t-test

**Table 2:** Correlations between right 2D:4D, left 2D:4D, CRT, CRT-2, and SVO angle in men and women.

Correlations								
	Males				Females			
	1	2	3	4	1	2	3	4
1. Right 2D:4D								
2. Left 2D:4D	.702^c^				.730^c^			
	(n = 118)				(n = 83)			
3. CRT	.038	-.046			.034	-.035		
	(n = 121)	(n = 123)			(n = 85)	(n = 87)		
4. CRT-2	-.078	-.112	.531^c^		-.100	.011	.466	
	(n = 121)	(n = 123)	(n = 127)		(n = 85)	(n = 87)	(n = 91)	
5. SVO angle	-.010	.036	-.012	.157^a^	-.004	-.026	.080	.153
	(n = 121)	(n = 123)	(n = 127)	(n = 127)	(n = 85)	(n = 87)	(n = 91)	(n = 91)

Notes:

^a^ p <.10;

^b^ p <.05;

^c^ p <.001

To test our main hypothesis, we regressed SVO angle on right 2D:4D (mean-centered), cognitive reflection score (mean-centered) and the corresponding interaction. Table 3 reports OLS regression results for SVO angle, disaggregated for (a) CRT-2 and CRT and (b) men and women.

**Table 3:** SVO angle as a function of 2D:4D and cognitive reflection.

CRT-2 (4 items)						
	(male)			(female)		
	Est	SE	p-value	Est	SE	p-value
2D:4D	-11.925	44.660	0.790	3.152	38.392	0.935
CRT-2	2.422	1.245	0.054	1.483	1.263	0.244
2D:4D*CRT-2	-67.078	41.144	0.106	-0.789	35.952	0.983
Constant	22.599	1.265	0.000	27.282	1.249	0.000
N		121			85	
R2		4.79%			1.75%	
CRT (3 items)						
	(male)			(female)		
	Est	SE	p-value	Est	SE	p-value
2D:4D	-8.053	45.062	0.859	-3.877	38.019	0.919
CRT	-1.218	1.283	0.344	0.668	1.150	0.563
2D:4D*CRT	-34.310	44.982	0.447	39.574	31.303	0.210
Constant	22.792	1.286	0.000	27.238	1.240	0.000
N		121			85	
R2		1.12%			2.25%	

**Fig 1:**
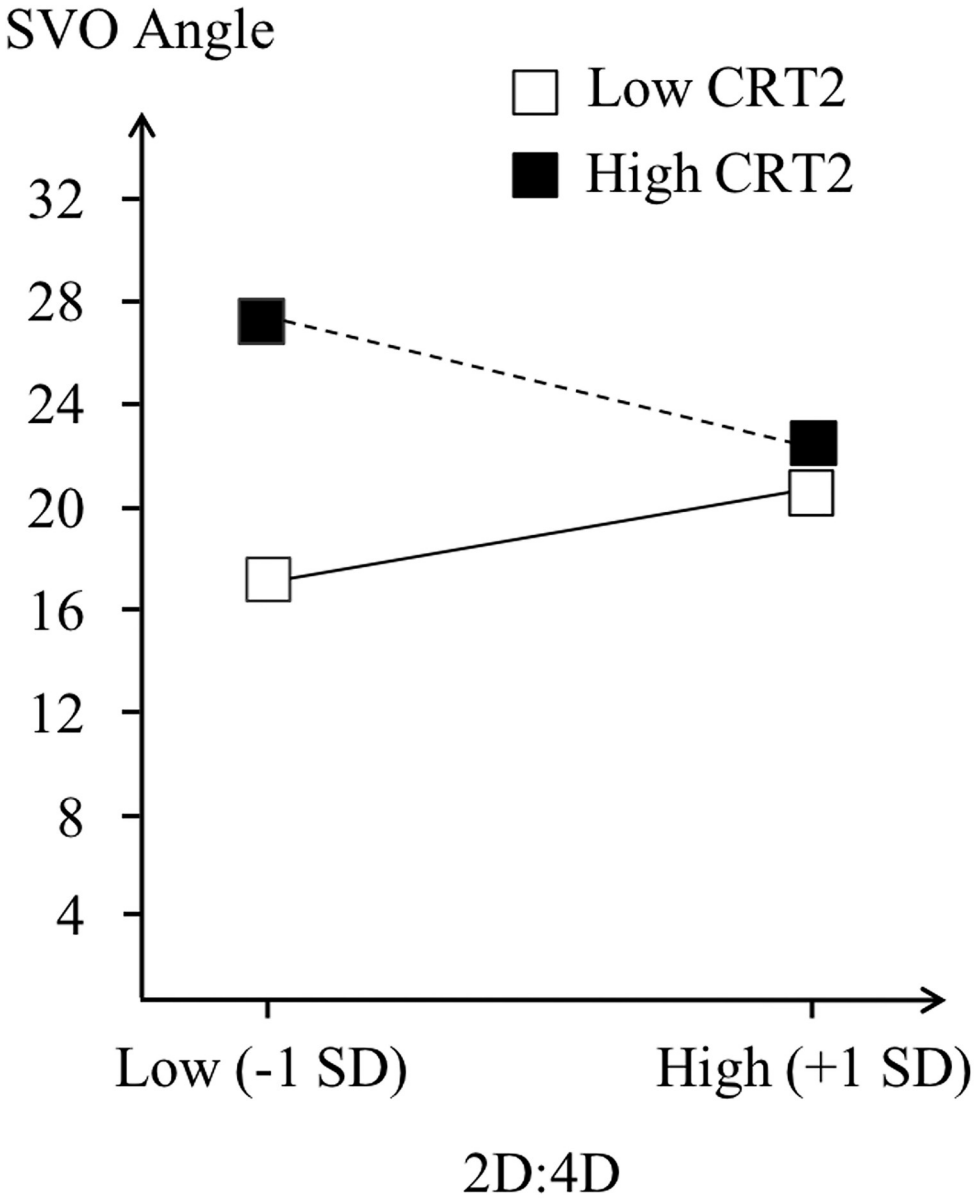
SVO as a function of 2D:4D and CRT-2 (Right-hand male sample).

**Fig 2:**
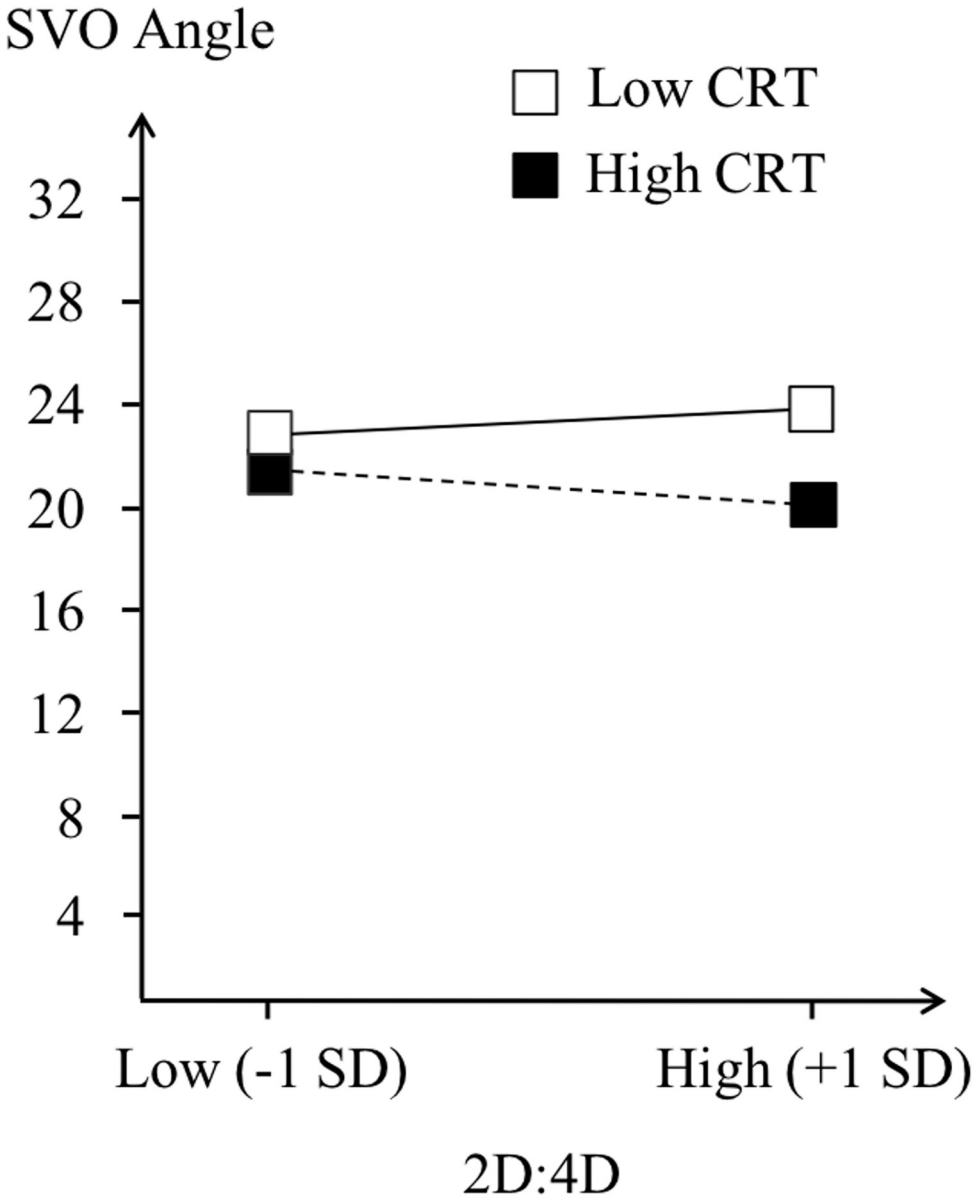
SVO as a function of 2D:4D and CRT (Right-hand male sample).

For men, the regression shows a positive effect of CRT-2 (β = 2.42, p *=* .054), but no significant effect of right 2D:4D (p = .789). Specifically, prosociality in men increases with greater levels of cognitive reflection. Importantly, the interaction term between the CRT-2 and right 2D:4D approaches marginal levels of significance *(*β = -67.08, p = .106). Therefore, we further explored the nature of this interaction via what is known as spotlight analysis or simple slopes analysis [58, 59]. Specifically, we estimated and tested the conditional effect of 2D:4D at different levels of CRT-2 and the conditional effect of CRT-2 at different levels of 2D:4D We followed the convention of testing conditional effects at plus or minus one standard deviation from the sample mean of an interacting variable [60]. Comparing the effect of 2D:4D on SVO angle at one standard deviation below and above the mean level of CRT-2 did not reveal any significant effects. However, the effect of CRT-2 on SVO angle varied at one standard deviation below and above the mean level of right 2D:4D. The results show a significant positive effect of CRT-2 for male participants with low right 2D:4D *(*β = 4.36, p = .017), but not for those with high right 2D:4D *(*β = .48, p = .768; see Fig 1). In other words, for male participants with low 2D:4D, the higher (lower) the cognitive reflection, the more (less) prosocial behavior they exhibit. However, for male participants with high 2D:4D, cognitive reflection did not affect their behavior. Interestingly, a similar regression analysis including the original CRT measure does not reveal any significant effects (all ps >.344; see Fig 2). For women, both regression analyses reveal non-significant results, either involving CRT-2 (all ps >.244) or CRT (all ps >.209).

As a follow up on these results, we ran further regression analyses in order to explore the possibility that the effect of cognitive reflection on prosocial behavior changes with the CRT measure being used. Thus, we regressed SVO angle on CRT (mean-centered), gender (contrast coded as -1 = male and 1 = female) and the corresponding interaction. Table 4 reports the results for SVO angle, disaggregated for CRT-2 and CRT.

**Table 4:** Social value orientation as a function of cognitive reflection and gender.

CRT-2 (4 items)			
	Est	SE	p-value
CRT-2	1.969	0.862	0.023
Gender	2.746	0.891	0.002
CRT-2*Gender	-0.211	0.881	0.811
Constant	24.582	0.878	0.000
N		218	
R2		5.99%	
CRT (3 items)			
	Est	SE	p-value
CRT	0.249	0.842	0.768
Gender	2.677	0.933	0.005
CRT*Gender	0.498	0.848	0.558
Constant	24.747	0.917	0.000
N		218	
R2		3.85%	

In both cases, the regression shows a positive effect of gender (β = 2.75, p *=* .002 and β = 2.68, p *=* .005, respectively), suggesting that women are more prosocial than men. More interestingly, we observe that CRT-2 significantly predicts prosociality (β = 1.97, p *=* .023) whereas CRT does not have any significant effect on prosocial behavior (β = .25, p *=* .768). The interaction between cognitive reflection and gender is not significant in both cases. These results corroborate our previous findings in the sense that CRT-2 and CRT differently relate to prosociality. The observed difference between the two measures of cognitive reflection is in line with previous research showing that CRT-2 is a better predictor of behavior than CRT is [54].

## Discussion

The current study is of importance for the growing literature on the biological foundations of prosocial behavior. Given the mixed findings currently reported in literature on how 2D:4D relates to prosocial behavior, more systematic investigation is needed to understand the relationship between 2D:4D and prosociality. Moreover, it is at least as important to focus on theoretically plausible moderators and to set up specific studies to understand if specific associations with 2D:4D emerge under particular circumstances. Therefore, the present study tested the possible moderating effect of cognitive reflection (a marker for intuitive vs. deliberative decision making) on the relationship between 2D:4D and prosociality. Further, the study procedure also allowed us to explore the potential direct relationship between cognitive reflection (using two separate measures, CRT and CRT-2) and prosociality (using the validated SVO slider measure). Speaking towards the reliability of our findings, we replicate previous findings showing (a) a sex difference in social value orientation [56], (b) a sex difference in performance on the classic CRT, which is attenuated for the CRT-2 [52] (c) a stronger sex difference in right 2D:4D than in left 2D:4D [6].

Our confirmatory analysis did not provide collective evidence for a moderating effect of cognitive reflection on the relationship between 2D:4D and SVO. This raises the question whether previously observed relationships between 2D:4D and social preferences or cognitive reflection are just type I errors or if the relationships that are reported in literature depend on other (omitted) variables. At least, null results are often not written up and therefore much (absence of) evidence is unobserved [61], despite the potential detrimental impact on scientific progress in the particular domain of interest. However, as suggested before, it is important to realize that different contextual factors may be of importance for a relationship with 2D:4D to occur. Recently, the findings on the relations reported between 2D:4D and risk taking, dominance and aggression were re-interpreted from the perspective that the particular behavior at hand in a specific context needs to be status relevant for a relationship to arise [26]. Therefore, the status relevance of the context at hand may be one interesting avenue for future research to explore relations between 2D:4D and prosociality.

Despite a lack of collective evidence with regard to our main hypothesis that cognitive reflection moderates the relationship between 2D:4D and prosociality, the interaction between right 2D:4D and CRT-2 on prosociality in men approaching significance yields further insights. Specifically, the effect of CRT-2 on prosociality in high and low 2D:4D men differs such that for low 2D:4D men, the more intuitive they are, the less prosocial they become, whereas for high 2D:4D men the amount of cognitive reflection does not affect their prosociality. This observation is consistent with our main hypothesis that low 2D:4D people may become less prosocial or more selfish when they make intuitive decisions. As this specific finding awaits confirmation in a follow-up research, caution is needed in the interpretation of this tentative result. That said, it is remarkable to see that this effect only seems to emerge (a) with the CRT-2 measure (b) in right 2D:4D measurements and (c) in men. If reliable, the question arises why we only found (at least part of) our expected pattern of results under these specific conditions. First, it is important to be aware that the CRT-2 measure is less reliant on numeracy than the original CRT [52]. The original CRT to some extent has been criticized to reflect numerical skills than deliberation [53]. Therefore, given that numerical skills by themselves may influence how people respond in a ‘numeric’ resource allocation paradigm such as SVO, this may have weakened potential relationship between the classic CRT and SVO. Furthermore, recent research provides evidence that CRT-2 behaves more in line with other measures of deliberative thinking and has superior predictive value over CRT [54]. Second, given that the gender difference is typically larger in the right than left 2D:4D, right 2D:4D has been considered a better indicator of prenatal androgenisation [6]. If anything, our results are at least consistent with this consideration.

In line with our pattern of results, different studies focusing on dominance-related outcomes observe stronger effects in men then women (e.g. relations between aggression and 2D:4D have been reported to be stronger in men than women [12, 62–64]). Considering that our prosociality measure is about the division of limited resources between oneself and another, one may consider the selfish behavior as an expression of dispositional dominance. In that case, the present data could suggest that the relation between dispositional dominance and 2D:4D in men may not necessarily arise because of the activation of the dominance system [14], but rather because of a reliance on intuition in challenge situations. However, as this suggestion is highly speculative, it is worthwile to first provide stronger evidence for this moderation between intuition and male 2D:4D on prosociality in a confirmatory study before exploring this avenue further.

Finally, it is important to be aware that our prosociality measurement is not incentivized, which may explain the collective lack of evidence for our main hypothesis. Whereas incentivization seems to be crucial to observe relationships between 2D:4D and financial risk taking [15], the same may hold for prosocial decision making. However, the literature on 2D:4D and prosocial decision making does not hint at a similar moderating role of incentivization on potential relationships between prosociality and 2D:4D. Moreover, in line with both our tentative results in men as well as the reasoning developed above, 2D:4D may not predict risk taking with real monetary incentives because of its potential status relevance (cf. [26]), but rather because of its increased reliance on intuition when taking specific riskful decisions with real (compared to fictitious) financial consequences. Again, this hypothesis awaits empirical testing and is a potential avenue for further research.

When we focus on the direct relationship between cognitive reflection and prosociality, we observe that CRT-2 (but not the CRT) positively predicts prosociality. Given that CRT-2 is likely to be a more reliable measurement of deliberative thinking (see above) our findings seem to be in support of a reflective, but not a heuristic, model of prosociality.

Summarized, our study highlights how important it remains to re-interpret many 2D:4D findings from a contextual perspective and to test and report both hypotheses and results irrespective of significance levels. While it is important to be aware that we did not provide supportive evidence in our confirmatory test of our main hypothesis, this did not prohibit us to explore the data further and provide a post hoc interpretation of the patterns of results. We find this a fruitful approach as long as this is clearly communicated in the paper. As such, we followed this approach and clearly distinguished between confirmatory and post hoc, exploratory research findings. In the end, this allows us to get more reliable insights into which factors may be more or less likely to be of importance in studying the relationship between 2D:4D and prosociality (or any other behavior) as only then the true role of 2D:4D in (economic) decision making can be discovered. Furthermore, our study results point to a positive association between deliberation and prosociality. In this regard, our findings suggest that it remains important to further examine the effect of cognitive deliberation on prosocial decision making in different settings and with different operationalizations of the constructs at hand. Finally, we would like to allocate some attention to the fact that the literature on 2D:4D has suffered from interpretations of findings that are limited to one hand, but are not replicated in the other hand. If anything, the more pronounced sex difference in right 2D:4D provides support to focus on only right 2D:4D (and to make that choice before the start of the study) or if focusing on both measurements, the observed effects should be replicated in both hands (and findings always reported if this choice had been made before the start of the study). Still, more direct replication studies are needed to test the robustness of these results regardless of which hand they pertain to. We speculate that right 2D:4D effects will be more easily replicated than left 2D:4D effects.

## Conclusions

In general, the current study does not provide evidence for the main hypothesis that the relationship between 2D:4D and social preferences may be influenced by people’s cognitive style. At the same time we urge for a confirmatory test of our observed pattern of results in men. At least, previous findings on sex differences in (a) right vs left 2D:4D, (b) CRT vs CRT-2 and (c) SVO are replicated and therefore speak towards the reliability of the results. Given the multitude of inconsistent findings and the omission of potentially important variables in the study on the connection between 2D:4D and social preferences, it is important to report studies that focus on both direct and moderating effects, irrespective of the significance of results. Therefore, the current study is of importance to the field. Finally, the relationship between CRT-2 and prosociality deserves further attention in future research.

## Supporting information

S1 File Supplementary materials. (DOCX)

S2 File The supporting dataset file. (SAV)

S1 Table Social value orientation as a function of 2D:4D and cognitive reflection (Left-hand sample). (DOCX)
