# Supplementary material for: Cognitive reflection, 2D:4D and social value orientation
Source: PLoS One. 2019 Feb 22;14(2):e0212767. doi: 10.1371/journal.pone.0212767 (PMC6386376; doi:10.1371/journal.pone.0212767)
Supplement: S1 File — (DOCX) [file pone.0212767.s001.docx]

**SUPPLEMENTARY MATERIALS**

**Secondary Items of the SVO Slider Measure**

From the responses to the six primary items of the SVO measure, we compute the SVO angle for each participant (as incorporated in the main text). Based on this SVO angle, we are able to identify different personality types in the data [38, 40]. As such, 2 participants are identified as altruistic (2 men) and 129 as prosocial (65 men, 64 women), whereas 87 are considered individualistic (60 men, 27 women) and none competitive.

The remaining nine secondary items are set up to determine which motive is more important to act prosocially. The responses of individualistic, competitive and altruistic types on the secondary items are not meaningful in that case. Therefore, the analysis of the secondary items only applies to individuals identified as ‘prosocial type’ [40]. Subsequently, a ‘Joint Gain Maximization’ index is calculated for only these participants that make consistent prosocial allocations in the primary and secondary items (as recommended by Murphy, Ackermann and Handgraaf [40]). This results in an index that ranges between 0 (choice allocation perfectly consistent with inequality aversion) and 1 (choice allocation perfectly consistent with a preference for joint gain maximization). An exploratory analysis provides evidence for a sex difference (t(106)=2.86; p = .005) showing that men (M= .32) are more driven by joint gain maximization than are women (M = .20). Given (a) the limited power of analyses within a gender specific sample due to the dropout of participants and (b) potential selection bias due to the positive relation between CRT-2 and SVO angle (accordingly, prosocial score marg. significantly higher on CRT-2 than individualistic types, *p* = .09), results from analyses with this measure are not meaningful. Still, these data are all available in our supplementary dataset.
