# Supplementary material for: Cognitive reflection, 2D:4D and social value orientation
Source: PLoS One. 2019 Feb 22;14(2):e0212767. doi: 10.1371/journal.pone.0212767 (PMC6386376; doi:10.1371/journal.pone.0212767)
Supplement: S1 Table — (DOCX) [file pone.0212767.s003.docx]

**S1 Table. Social Value Orientation as a function of 2D:4D and cognitive reflection (Left-hand Sample).**

| CRT-2 (4 items) |  |  |  |  |  |  |  |
| --- | --- | --- | --- | --- | --- | --- | --- |
|  | (male) |  |  |  | (female) |  |  |
|  | Est | SE | p-value |  | Est | SE | p-value |
| 2D:4D | 27.520 | 47.022 | 0.560 |  | -6.731 | 44.180 | 0.879 |
| CRT-2 | 2.091 | 1.236 | 0.093 |  | 1.665 | 1.219 | 0.176 |
| 2D:4D*CRT-2 | -1.016 | 44.218 | 0.982 |  | 25.121 | 43.737 | 0.567 |
| Constant | 22.380 | 1.293 | 0.000 |  | 27.172 | 1.213 | 0.000 |
| N |  | 123 |  |  |  | 87 |  |
| R2 |  | 2.48% |  |  |  | 2.49% |  |
|  |  |  |  |  |  |  |  |
| CRT (3 items) |  |  |  |  |  |  |  |
|  | (male) |  |  |  | (female) |  |  |
|  | Est | SE | p-value |  | Est | SE | p-value |
| 2D:4D | 18.405 | 47.349 | 0.698 |  | -7.945 | 44.371 | 0.858 |
| CRT | -0.151 | 1.261 | 0.905 |  | 0.519 | 1.162 | 0.656 |
| 2D:4D*CRT | -7.496 | 46.690 | 0.873 |  | 8.574 | 39.891 | 0.830 |
| Constant | 22.374 | 1.302 | 0.000 |  | 27.204 | 1.226 | 0.000 |
| N |  | 123 |  |  |  | 87 |  |
| R2 |  | 0.17% |  |  |  | 0.34% |  |
